# The Effect of Anxiety and Autism Symptom Severity on Restricted and Repetitive Behaviors Over Time in Children with Fragile X Syndrome

**DOI:** 10.21203/rs.3.rs-3353765/v1

**Published:** 2023-11-02

**Authors:** Lauren Moskowitz, Elizabeth Will, Conner Black, Jane Roberts

**Affiliations:** St. John’s University; University of South Carolina; NewYork-Presbyterian Weill Cornell Medical Center; University of South Carolina

**Keywords:** fragile X syndrome, restricted repetitive behaviors, autism, anxiety

## Abstract

**Background:**

Restricted and repetitive behaviors (RRBs) are highly prevalent and reduce function in individuals with fragile X syndrome (FXS). As transdiagnostic features of intellectual disability, elevated rates of RRBs in FXS could represent various underlying known co-occurring conditions in FXS such as anxiety or autism spectrum disorder (ASD), yet this distinction has not been investigated. Further, delineating whether RRBs are more indicative of anxiety or ASD in FXS may clarify phenotypic profiles within FXS and improve differential assessment.

**Methods:**

We longitudinally examined the potentially independent or multiplicative effect of ASD and anxiety symptom severity on RRBs in 60 children with FXS. Anxiety was measured using the Child Behavior Checklist (CBCL), ASD severity was measured using the Childhood Autism Rating Scale (CARS), and RRBs were measured using The Repetitive Behavior Scale – Revised (RBS-R). We estimated a series of moderated regression models with anxiety and ASD symptoms at the initial assessment (Time 1) as predictors of RRBs at the outcome assessment two years later (Time 2), along with an anxiety-by-ASD interaction term to determine the potential multiplicative effect of these co-occurring conditions on RRBs.

**Results:**

Results identified a significant interaction between ASD and anxiety symptom severity at the initial assessment that predicted elevated sensory-motor RRBs two years later. Increased sensory-motor RRBs were predicted by elevated ASD symptoms only when anxiety symptom severity was low. Likewise, increased sensory-motor RRBs were predicted by elevated anxiety symptoms only when ASD symptom severity was low. Interestingly, this relationship was isolated to Sensory-Motor RRBs, with evidence that it could also apply to total RRBs.

**Conclusions:**

Findings suggest that ASD and anxiety exert independent and differential effects on Sensory-Motor RRBs when at high severity levels and a multiplicative effect when at moderate levels.

Fragile X syndrome (FXS) is the most common inherited cause of intellectual disability (ID) and the leading known single-gene cause of autism, with rates of FXS occurring in approximately 1 in 7,000 males and 1 in 11,000 females (Hunter et al., 2014). FXS is a single-gene disorder caused by a CGG repeat expansion on the *Fragile X Messenger Ribonucleoprotein 1 (FMR1)* gene on the X chromosome, resulting in reduced production of *Fragile X Messenger Ribonucleoprotein Protein (FMRP)*, which is a protein that is crucial to brain development (Hagerman et al., 2012). Typically, males with FXS are more severely affected than females due to the protective or compensatory effect of the second unaffected X chromosome in females (Hagerman & Hagerman, 2002). FXS is characterized by a complex behavioral phenotype that includes ID (Boyle & Kaufmann, 2010); impairments in language and communication (Abbeduto et al., 2007); anxiety (Cordeiro et al., 2011; Ezell et al. 2019); elevated physiological arousal (Hogan et al., 2021; Klusek et al., 2013); self-injurious and aggressive behavior (Crawford et al., 2019); and autistic-like symptoms such as social avoidance, poor eye contact/gaze aversion (Cohen et al., 1989; Roberts et al., 2019a, b) and restricted and repetitive behaviors (RRBs; e.g., hand-flapping, verbal perseveration; Moss et al., 2009). In addition to these discrete phenotypic features, individuals with FXS are at increased risk for specific comorbid disorders, most notably anxiety and autism spectrum disorder (ASD). There is substantial overlap in symptomatology specific to ASD and anxiety, as well as symptomology that could be independent of ASD and anxiety as part of the general FXS phenotype. Thus, the FXS phenotype is highly complex and includes features of multiple co-morbid conditions that emerge and likely interact across development. The complexity of the FXS phenotype poses substantial challenges for accurate and early diagnosis critical to optimizing outcomes in children with FXS. As such, studies focused on disentangling the relationships among multiple features of the FXS phenotype employing a developmental framework are critical. The present study focused on delineating the role of autism and anxiety features on RRBs in FXS over time.

## Restricted and repetitive behaviors in FXS

Restricted and repetitive behaviors (RRBs) are nearly universal in FXS (Moss et al., 2009). RRBs are a broad class of behaviors that includes *sensory-motor behaviors* (e.g., hand-flapping), *self-injurious behavior* (*SIB*) (e.g., biting oneself), *restricted interests* (e.g., preoccupation with one subject or activity), *compulsive behavior* (e.g., arranging/ordering, closing open doors), and *ritualistic/sameness behavior* (“insistence on sameness”; e.g., resists changing activities). Although RRBs can be considered a transdiagnostic or common marker for various neurological and neurodevelopmental disorders (Kästel et al., 2021; Martínez-González & Piqueras, 2018), RRBs are elevated in FXS in comparison to other genetic syndromes including those associated with ID (Moss et al., 2009). Up to 98% of males with FXS display repetitive motor movements/stereotypy (Hessl et al., 2008), 79% of males display SIB (Hessl et al., 2008), 73% of males display repetitive questions, repetitive speech, or restricted conversation about the future (Woodcock et al., 2009), and 72% of males and 55% of females exhibit compulsive behavior (Hall et al., 2008). Given that most males with FXS show a more severe clinical presentation than females, research on RRBs in females with FXS is lacking, with the limited research showing that females with FXS are rated as exhibiting less severe RRBs than males with FXS, with the exception of Compulsive behaviors (Moskowitz et al., 2020; Reisinger et al., 2020). Nevertheless, across both males and females with FXS, higher rates of RRBs are associated with poorer outcomes in FXS including greater cognitive impairment, lower adaptive behavior skills, and higher rates of problem behavior (Reisinger et al., 2020). Accordingly, it is essential to identify the key factors contributing to RRBs in FXS to attenuate their potential associated consequences; yet, despite the high prevalence of RRBs in FXS overall, these currently remain poorly understood.

RRBs can often be classified into “lower-order” types that involve repetitive motor movements or self-injury, as well as into “higher-order” types that are more complex and cognitively mediated, such as ritualistic/sameness behavior or compulsive behavior or restricted interests (Bishop et al., 2006). Although this classification can be useful in characterizing the form and topography of RRBs, these two broad categories may potentially obscure more nuanced differences in the underlying function or contributing mechanisms (Moskowitz et al., 2020; Oakes et al., 2016). Many core and associated features of the FXS phenotype have been implicated in the presence and severity of RRBs, regardless of whether higher- or lower-order. For instance, nonverbal IQ was shown to predict RRB severity in individuals with FXS in some studies (Abbeduto et al., 2009; Oakes et al., 2016), though it only predicted compulsive behavior in Moskowitz et al. (2020). Further, Abbeduto and colleagues (2019) found that RRB severity was best predictbed by psychiatric symptoms consistent with anxiety, social avoidance, and manic/hyperactive behavior in adolescent and young adult males with FXS. The substantial overlap in symptomatology between anxiety, ASD, and the FXS phenotype introduces complexity in delineating the specific underlying function of RRBs in FXS. The possibility that anxiety may drive autistic features in FXS (Thurman et al., 2014; McDuffie et al., 2010) exacerbates the FXS phenotypic complexity and poses a challenge in disentangling the various factors that may contribute to these clinical phenotypic features. While anxiety has been found to predict RRBs in males with FXS (Abbeduto et al., 2019; Oakes et al., 2016), no studies have examined the longitudinal relationship between anxiety and RRBs in both males and females with FXS while also taking autism symptomatology into account, as the present study does. Considering the high but variable presentation of RRBs in FXS and their association with greater levels of functional impairment (Reisinger et al., 2020), distinguishing the specific contributing factors of various RRB subtypes can better inform phenotypic characterization and targeted treatment (Oakes et al., 2016).

## Autism in FXS

ASD is highly prevalent in FXS and may differentially or multiplicatively account for the presence or severity of RRBs. Specifically, 54–80% of males and 14–41% of females meet diagnostic criteria for ASD (e.g., Abbeduto et al., 2019; Klusek et al., 2014; Lee et al., 2016). More recently, 60.7% of preschoolers with FXS were found to meet diagnostic criteria for ASD (73% of boys and 28.6% of girls), even when employing expert clinical judgement to distinguish ASD from developmental delay, anxiety, or ADHD (Roberts et al., 2020), reducing the potential for inflated prevalence which can occur when using cutoff scores in isolation (Zheng et al., 2023). Because RRBs are a core diagnostic domain for ASD (APA, 2013), the presence of comorbid ASD in FXS may also contribute to elevated RRBs independent of, or in conjunction with, co-occurring anxiety.

Prior evidence has identified behavior-specific associations between RRBs and ASD in FXS. Specifically, some studies show that individuals with FXS and comorbid ASD are no more likely to show certain types of RRBs (e.g., compulsive behavior and self-injury) than those with FXS without ASD (Hall et al., 2008; Symons et al., 2003), suggesting that ASD symptomatology may *not* be the primary driver of RRBs in FXS (Oakes et al., 2016). However, other types of RRBs (sensory-motor behaviors, restricted interests, behavioral inflexibility) *do* appear at higher rates in children with comorbid FXS+ASD compared to children with FXS without ASD (Harrop et al., 2021, Reisinger et al., 2020). Recent evidence also shows that the severity of RRB subtypes in early development differentiates the presence of co-occurring autism in FXS from FXS-only in middle childhood. Specifically, severity of sensory-motor behavior, restricted interests, self-injury, and compulsive behavior all significantly predicted later ASD *diagnostic* outcomes in children with FXS (Roberts et al., 2020), suggesting that the underlying existence of ASD as a developmental liability in FXS may contribute to RRBs. Thus, the severity of ASD features in FXS may either contribute to or exacerbate existing RRBs in FXS.

However, it is important to note that, although compulsive behavior, insistence-on-sameness behaviors, and total level of RRBs were found to significantly relate to ASD symptomology in males with FXS (Moss et al., 2009), this effect was specific to just one item (i.e., only “just right” behavior correlated with autism symptoms). Coupled with the transdiagnostic nature of RRBs within ID, this evidence suggests that, in FXS, alternative mechanisms – such as anxiety – may account for RRBs in FXS in addition to (or instead of) ASD phenomenology (Moss et al., 2009).

## Anxiety in FXS

Anxiety is one of the most prominent characteristics of FXS (Cordeiro et al., 2011) and the most common symptom treated in FXS (Bailey et al., 2012). Indeed, individuals with FXS are more likely to meet criteria for anxiety disorders than other ID groups or the general population (Cordeiro et al., 2011). Approximately 52–86% of males with FXS and 77% of females with FXS aged 5 to 26 years meet diagnostic criteria for at least one anxiety disorder (Cordeiro et al., 2011; Ezell et al., 2019), with even 39% of preschoolers with FXS meeting criteria for at least one anxiety disorder (Roberts et al., 2020), though rates differ based on age, sex, cognitive ability, and the measure(s) used.

Due to the large incidence of co-occurring anxiety in FXS, it is possible that RRBs may function as a coping strategy for anxiety in FXS – that is, *anxiety may cause or at least contribute to RRBs in FXS*. Indeed, at least one type of RRB – verbal perseveration, considered a hallmark of FXS – has often been attributed to the excessive anxiety that is a classic feature of the FXS behavioral phenotype (Belser & Sudhalter, 1995; Friedman et al., 2018; Martin et al., 2012; Murphy & Abbeduto, 2007). In support of this notion, Crawford and colleagues (2023) recently found that stronger anxiety/stress responses were associated with heightened repetitive language in males with FXS. Further, evidence suggests a long-term association between anxiety and RRBs in males with FXS (Oakes et al., 2016). Specifically, early anxiety predicted Restricted Interests, Compulsive behaviors, and Ritualistic/Sameness RRBs 18 months later in boys with FXS, suggesting that anxiety could be a causal or at least contributing factor for some types of RRBs in youth with FXS (Oakes et al., 2016). For instance, 76% of boys with FXS were reported by their parents to exhibit repetitive questions following changes to routines or expectations, with parents reporting that most of these children already knew the answers to the questions that were asked (Woodcock et al., 2009). It is thus possible that RRBs such as repetitive questioning and other types of verbal perseveration observed frequently in FXS may increase predictability (given that the child already knows the answers), thereby reducing anxiety. This repetitive questioning is likely reinforcing to the child because it allows them to escape or avoid the aversive state of anxiety due to changes in routine, thus contributing to further repetitive questioning (Woodcock et al., 2009). However, as with co-occurring ASD in FXS, the phenotypic complexity and multitude of co-morbid conditions in FXS pose challenges to identifying underlying contributors of clinical features such as RRBs, which is essential for improving differential assessment and diagnosis.

## Overlapping Features

Therefore, although anxiety may contribute to some RRBs in FXS, it is also likely that certain RRBs may *not* function as a manifestation of anxiety, particularly in the context of the phenotypic complexity of FXS and considering the transdiagnostic nature of RRBs in ID (Symons et al., 2005). Accordingly, other factors may account for RRBs in FXS, including co-occurring ASD in isolation, or the interaction between anxiety and ASD in FXS (Abbeduto et al., 2014; Roberts et al., 2018, 2020; Smith et al., 2021). Evidence for this interaction appears in non-syndromic ASD (nsASD), where, despite RRBs being a core diagnostic feature of ASD, they are associated with increased anxiety symptom severity in this population (Rodgers et al., 2012; Russell et al., 2019). Specifically, in youth with nsASD, Rodgers et al. (2012) found that those with high anxiety displayed more RRBs than those without anxiety. While an association between general anxiety and overall RRBs has been demonstrated in nsASD, there is also evidence for anxiety relating to specific RRB subtypes (Russell et al., 2019). For example, anxiety significantly relates to insistence-on-sameness behaviors in children and adolescents with nsASD as well as the severity of overall RRBs (Lidstone et al., 2014; Russell et al., 2019).

This pattern of shared (or possibly distinct) mechanistic roles of anxiety or ASD symptomatology in the severity of RRBs may also exist in FXS. For instance, certain anxiety disorders, including Specific Phobia and Social Anxiety, appear at higher rates in individuals with both FXS+ASD versus those without ASD (Cordeiro et al., 2011), suggesting that when anxiety and ASD co-occur together in FXS, they may exacerbate features of the other. Indeed, comorbid anxiety is associated with increased ASD severity in males with FXS (Talisa et al., 2014). Further, in addition to leading to increased RRBs, anxiety can present as poor eye contact and social avoidance in FXS (Klusek et al., 2014), which can alternatively translate as greater social impairment associated with ASD (Roberts et al., 2019). However, additional evidence has differentiated ASD severity from anxiety in adolescent males with FXS (Ezell et al., 2019), as well as in both adults and across childhood (Crawford et al., 2020; Roberts et al., 2019a; 2020). These conflicting findings may be due to differences in the sample (sex, age, IQ), whether assessment of anxiety accounted for intellectual and communication impairments, and that many measures lack the sensitivity necessary to distinguish overlapping symptomatology of anxiety and ASD in FXS. Regardless, the substantial overlap in phenotypic features across anxiety, autism, and FXS complicates early and differential diagnosis, yet the nature of ASD or anxiety as functions of RRBs in FXS remains unclear considering contradictory evidence and the lack of a truly developmental approach in prior research.

## The Present Study

The emergence of anxiety or ASD in FXS shapes developmental trajectories and outcomes for children with FXS, regardless of how each may relate to RRBs. Yet, because RRB subtypes may present differently in FXS in the context of increased ASD severity (Crawford et al., 2018), or may reflect increased anxiety independent of ASD (Abbeduto et al., 2019; Belser & Sudhalter, 1995; Woodcock et al., 2009), disentangling these features as potential mechanisms of RRBs is important for accurate clinical assessment and targeted treatment for youth with FXS. The high prevalence and degree of impairment associated with RRBs in FXS warrants an increased understanding of the functional role anxiety or ASD plays in these outcomes within a developmental framework. This effort is particularly critical to effectively identify the timing and targets for intervention. Thus, using a prospective longitudinal design, the present study documents the potentially independent or multiplicative effect of ASD symptom severity and anxiety on the severity of RRB outcomes in both males and females with FXS across development. Specifically, we examine the potential interaction between anxiety and ASD symptomatology at an initial assessment in predicting RRB outcomes 2 years later, controlling for both age and level of RRBs at the initial assessment. Further, we examine individual subtypes of RRBs along with overall RRBs.

## Methods

### Participants

Data for this study was drawn from two independent, but related, studies [withheld for review] as part of larger prospective longitudinal studies on the early development of children with FXS. While multiple papers have been published from these studies (Roberts et al., 2019, 2020; Smith et al., 2021; Wall et al, 2019), this paper represents a unique set of questions that have never been posed. The current study included data from 60 youth diagnosed with FXS (see Table 1 for descriptive statistics) who had complete data for two assessments. The initial assessment was conducted between 1 and 14 years of age with a mean of 4.95 years (*SD* = 4.07, Range: 1–13.67). The outcome assessment was conducted approximately two years later with a mean age of 7.69 (*SD* = 3.84, Range: 3.56–16.62). Diagnosis of FXS was confirmed through genetic report.

### Measures

#### Measurement of restrictive and repetitive behaviors.

The Repetitive Behavior Scale – Revised (RBS-R; Bodfish et al., 2000) was utilized to measure the presence and prevalence of RRBs. The RBS-R is 43-item parent-report questionnaire that assesses RRBs across six subscales on a scale from 0 (behavior does not occur) to 3 (behavior occurs and is a severe problem). The RBS-R was normed on individuals with intellectual disability, and multiple other studies have created different factor structures of repetitive behaviors in individuals with ASD (Bishop et al., 2013). Consistent with prior work in autism and FXS (Oakes et al., 2016; Moskowitz et al., 2020), we employed the five-factor structure from Bishop et al. (2013) that includes: Sensory-Motor behaviors, Restricted Interests, Self-Injurious Behavior, Compulsive Behavior, and Ritualistic/Sameness. These five factors are very similar to the six RBS-R subscales, with the biggest change being that the two subscales of Compulsive and Ritualistic behavior are essentially combined into one factor. This 5-factor structure made more sense conceptually than using the 6 subscales, given that many of the items from the RBS-R Ritualistic Behavior subscale (e.g., insists on certain pre-bedtime routines, insists on eating/drinking only certain things, insists on taking certain routes/paths) appear to overlap with items from the RBS-R Sameness subscale (e.g., insists on walking in a particular pattern, insists on same routine, household, school, or work schedule every day).

#### Cognitive ability.

Cognitive ability was measured via one of three measures depending on the participant’s age at the time of assessment. The *Mullen Scales of Early Learning* (*MSEL*; Mullen, 1995), *Differential Abilities Scale – Second Edition* (*DAS*; Elliot, 2007), or *Leiter-R* (Roid & Miller, 1997) were utilized to assess the participants’ current cognitive ability.

The MSEL (Mullen, 1995) is a developmental measure utilized to assess cognitive abilities in children between 0 and 68 months of age. The MSEL assesses cognitive abilities across five domains: Gross Motor, Fine Motor, Visual Reception, Receptive Language, and Expressive Language. Nonverbal cognition can be measured by taking a composition of Fine Motor and Visual Reception. This measure was standardized across 1849 children and demonstrated satisfactory test-retest reliability, internal consistency, and concurrent validity.

The DAS (Elliot, 2007) is a measure that assesses cognitive abilities in children between 2:6 and 17:11 years of age. The measure was standardized on a large sample of children (*N* = 3480), demonstrating excellent internal consistency, satisfactory test-retest reliability, and acceptable concurrent validity. The current study will utilize a nonverbal composite score that is calculated by using Matrices and Picture Similarities.

The Leiter-R (Roid & Miller, 1997) is an assessment tool used to assess nonverbal intelligence in children between 2 and 20 years 11 months of age. This measure was normed on a sample of 1719 children with acceptable internal consistency, test-retest reliability, and concurrent validity. The nonverbal composite is created using four subtests: Figure Ground, Form Completion, Sequential Order, and Repeated Patterns.

Due to the inherent differences between the MSEL, DAS, and Leiter-R, Z-scores were computed from nonverbal IQ composite scores to equate NVIQ estimates across measures.

#### Anxiety.

Anxiety symptoms were measured using the DSM Anxiety Problems scale of the Child Behavior Checklist (CBCL; Achenbach & Rescorla, 2001). The CBCL is a parent-report measure that assesses a variety of emotional and behavioral problems in children. Parents completed the version appropriate for their child’s age (CBCL 1.5–5 Years; CBCL 6–18 Years). The DSM Anxiety Problems scale was developed to assess a wide range of symptoms of anxiety including worries, fear of going to school, and clinging to adults. To increase the variability of the sample, raw scores were utilized instead of T scores. (Given the different number of items on the two CBCL scales, a mean score was calculated that reflected the total number of items on the respective scale. Specifically, the total raw score was divided by the total number of items on the age-appropriate scale.)

#### ASD Symptom Severity.

ASD symptom severity was assessed using the Childhood Autism Rating Scale (CARS; Schopler et al., 1988). The CARS is an observational rating scale completed by a consensus of multiple trained research team members that assess specific ASD features across 15 symptom areas. The Total Raw Score was used as a primary variable in analyses.

### Procedure

The institutional review board at both [withheld for review] and [withheld for review] provided ethical approval. Participants were recruited through local and national organizations serving FXS populations. Data collection occurred in either the research laboratory or the family’s home depending on family preference. Families were provided with monetary compensation and a brief developmental report at the completion of each time point.

### Data Analyses

All variables were examined for errors and non-normality, and no problems were identified. Descriptive statistics were calculated for all primary variables in the study and are presented in Table 1. The primary aim of the study was to determine whether ASD and anxiety symptom severity exert a mutual or distinct influence on RRBs over time. That is, does ASD or anxiety independently account for the presence of RRBs, or do they interact (exacerbate) one another in a manner that influences degree and severity of RRBs? Accordingly, we estimated a series of moderated regression models with anxiety and ASD symptoms at the initial assessment (Time 1) as predictors of RRBs at the outcome assessment (Time 3), along with an anxiety-by-ASD interaction term to determine the potential multiplicative effect of these co-occurring conditions on RRBs. Additionally, chronological age and RRBs at Time 1 were included as covariates in each model. Separate models were estimated for each of the five RRB factors: Sensory-motor, Restricted Interests, Self-Injury, Compulsive Behavior, and Ritualistic Sameness, as well as a sixth model for RBS Total Score.

## Results

To consider moderating effects between anxiety and ASD on later repetitive behaviors, each RRB factor was regressed on T1 anxiety and ASD severity, an interaction between these, and relevant covariates: T1 repetitive behavior factor and T1 chronological age. All model results are presented in Table 2.

For the model predicting outcomes in Sensory-Motor behavior, there was a statistically significant interaction between anxiety and ASD severity, indicating that, as one increases, the effect of the other on Sensory-Motor RRBs over time decreases (*b* = −0.40; *p* = 0.024). There were no significant effects for the interaction between anxiety and ASD in predicting Restricted Interests, Self-injury, Compulsive behavior, or Ritualistic/Sameness outcomes (see Table 2). The final model testing the interaction effect between anxiety and ASD severity on Total RBS outcomes revealed a trending association (*b* = −1.19, *p* = 0.081). This effect was consistent in that increased ASD severity over time was associated with a lessened effect of early anxiety on later total RRBs.

**Figure 1:**
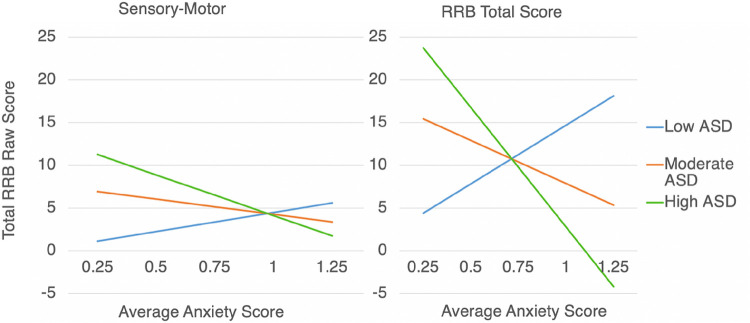
Interactions between ASD and Anxiety on Sensory Motor RRBs (Left) and Total RRB (Right) Outcomes *Note: Effects were trending towards significance for the Total RRBs model (p<0.10)

Figure 1 illustrates the interaction effects for Sensory-Motor RRBs and total RRBs over time. Interesting patterns emerged from the interactions such that, at low levels of either anxiety or ASD, the inverse (i.e., high ASD or high anxiety) accounted for elevated Sensory-Motor behaviors. That is, in the context of high anxiety, the effect of ASD severity on repetitive Sensory-Motor behaviors was lessened to the extent that high anxiety appeared to account for elevated levels of Sensory-Motor RRBs. Likewise, at high ASD severity levels but in the context of low anxiety, high ASD severity appeared to account for elevated levels of Sensory-Motor RRBs over time. Additionally, when anxiety and ASD were both estimated at moderate levels, Sensory-Motor RRBs also appeared moderate. Interaction effects for Total RRBs were similar.

## Discussion

Results of the present study suggest potentially distinct mechanisms of RRBs in youth with FXS. Our findings illustrate that elevated levels of Sensory-Motor RRBs are predicted by either low ASD symptom severity coupled with high anxiety or, conversely, by high ASD symptom severity coupled with low anxiety (see Fig. 1). Thus, we observed a “see-saw” effect, with elevated ASD symptom severity suppressing the effect of anxiety on Sensory-Motor RRBs and vice-versa (i.e., with elevated autism anxiety symptom severity suppressing the effect of ASD on Sensory-Motor RRBs) across children with FXS. Interestingly, this suggests that either anxiety *or* autism symptomatology can account for increased levels of Sensory-Motor RRBs over time independently of one another, even when controlling for early RRBs.

Our results differ somewhat from the findings of Oakes et al., (2016), who found that anxiety at the initial assessment in 6- to 10-year-old boys with FXS predicted Restricted Interests, Compulsive behaviors, and Ritualistic/Sameness RRBs approximately 18 months later. However, it is important to note that this study (Oakes et al., 2016) did not account for RRBs at the initial assessment, while we did so in the current study. Another reason for our differing findings is that some of the effects of anxiety in the present study are evidently solely accounted for by ASD; when levels of each (anxiety and ASD) are moderate, they are not well-differentiated. Therefore, accounting for the contribution of ASD when examining the relationship between anxiety and RRBs provides a more precise picture of how anxiety specifically impacts RRBs in youth with FXS over time. Our sample also included a wider age range (1–18 years) than Oakes et al., (2016) as well as both males and females. Given that the phenotypic profile of RRBs for females with FXS is distinct from that for males (Moskowitz et al., 2020), with males with FXS rated as having more Sensory-Motor behaviors and females with FXS rated as having more Compulsive behaviors (Reisinger et al., 2020), the inclusion of females in our study is also an important consideration for cross-study comparisons.

Our findings that, as ASD severity increased, there was less of an effect of early anxiety on later Sensory-Motor behaviors and overall RRBs (with trends towards significance for overall RRBs) – were somewhat unexpected, considering evidence of the strong co-occurrence between anxiety and ASD in FXS. However, experts in the field debate the validity of ASD diagnoses in FXS (Abbeduto et al., 2014; Roberts et al., 2020; see also Hall et al., 2010), with some positing that features of ASD are manifestations of co-occurring anxiety in FXS rather than ASD (e.g., Abbeduto et al., 2014; Belser & Sudhalter, 1995; Martin et al., 2012; Rajaratnam et al., 2020; Thurman et al., 2014). Verbal perseveration, for example, is an overlapping feature between anxiety and ASD, and has been hypothesized to account for an ASD diagnosis in FXS (Martin et al., 2012). Moreover, this specific RRB (verbal perseveration) may affect social communication and interaction, further inflating social impairments that may lead to an ASD diagnosis (Friedman et al., 2020; Martin et al. 2012). Arguably, RRBs as a feature of autistic symptomatology in FXS could manifest from attempts to reduce anxiety, rather than as actual evidence for co-occurring autism. In fact, some researchers even hypothesize that improving anxiety will facilitate improvements in autism severity in FXS (Rajaratnam et al., 2020). However, the results of our study indicate that the underlying source of RRBs – at least Sensory-Motor RRBs – in FXS varies as a function of the severity of anxiety and ASD symptomatology, suggesting that *either* ASD or anxiety may “drive” elevated levels of RRBs rather than both. Our findings align with other recent evidence supporting anxiety and ASD are two distinct disorders that may co-occur independently of or in conjunction with one another in the FXS phenotype (Crawford et al., 2017; Roberts et al., 2019a, 2020; Wall et al., 2019). Ultimately, the present findings provide further contribution to the field by recognizing the complex interplay of both anxiety and ASD symptoms in the prediction of RRB’s in FXS.

It appears that, based on the findings of the present study, for youth with more severe ASD, anxiety has less of an effect on later Sensory-Motor behaviors, and, to some extent, overall RRBs, and vice-versa (i.e., for youth with more severe anxiety, ASD symptom severity has less of an effect on Sensory-Motor RRBs, and to some extent, overall RRBs). This could be a measurement artifact due to “diagnostic overshadowing,” which refers to the propensity for parents or practitioners to attribute symptoms of anxiety to the autism itself (Reiss et al., 1982). In this way, symptoms of anxiety in youth with FXS might be overlooked or unnoticed because they are overshadowed by high levels of autism symptomatology. Conversely, it is also possible that high levels of anxiety could overshadow symptoms of ASD in FXS so that, for youth with higher levels of anxiety, autism symptoms are not as noticeable and therefore are *perceived* to have less of an impact on Sensory-Motor behaviors or overall RRBs.

In considering that anxiety and ASD may independently contribute to specific RRBs, as our findings suggest, this could mean there are two different mechanisms or pathways to Sensory-Motor RRBs in youth with FXS: one through anxiety, and the other through autism. As such, it is important to also consider the underlying function specific RRBs may serve. For instance, children with FXS may engage in motor stereotypies as a means of self-regulation or self-soothing to mitigate anxiety (Joosten et al., 2009; Russell et al., 2019), or as an enjoyable activity, as a means of “stimming” or ego syntonic behavior (Guertin et al., 2022). Using an applied behavior analysis (ABA) framework, RRBs may function to provide automatic negative reinforcement in the context of anxiety (reducing anxiety) or provide automatic positive reinforcement in the context of ASD (increasing pleasurable sensory input). Whereas Compulsive and Ritualistic/Sameness behaviors both likely function to avoid, escape, or reduce anxiety, Sensory-motor RRBs could serve both functions; some children may perform sensory-motor behaviors (e.g., body-rocking) to engage in calming activities when their anxiety or arousal levels become too high in order to bring them down to a manageable level, whereas others may engage in sensory-motor RRBs if their arousal levels are too low in order to bring them up to an optimal level.

Because different RRBs may serve different functions, treatment approaches should also differ accordingly to maximize treatment efficacy. This has significant implications for the development of specific behavioral interventions for children with FXS, which are already somewhat limited in application (but see Hall et al., 2020). Indeed, research has demonstrated that behavioral interventions for challenging behaviors in individuals with developmental disabilities are twice as likely to be effective if they are based on a functional assessment of the factors that evoke and maintain the behavior than if they are not (e.g., Carr et al.,1999). The substantial within-syndrome variability in the form and function of RRBs in FXS (Roberts et al., 2019) necessitates individualized assessment and intervention (Moskowitz & Jones, 2015).

### Restricted Interests, Compulsive, Self-injurious, and Ritualistic-Sameness Behavior

Although we found that, as ASD severity increases, the effect of early anxiety on later Sensory-Motor behaviors lessens, we did not find a significant main effect or interaction effect for anxiety and ASD predicting Restricted Interests, Compulsive behavior, Self-injury, or Ritualistic-sameness behavior. This is somewhat surprising for Compulsive and Ritualistic-sameness behavior, given previous cross-sectional findings that scores on the Compulsive behavior subscale, Insistence-on-sameness subscale, and total RRB scores were significantly correlated with autism symptomatology for males with FXS (Moss et al., 2009). However, there are several possible explanations for the lack of significant effects. First, it is possible that Sensory-Motor behaviors were more likely to show significant effects because they (along with Restricted Interests) are the most problematic types of RRBs in both males and females with FXS (Oakes et al., 2016; Reisinger et a., 2020). Second, it is possible that we did not find significant effects for these subtypes because the majority of research examining RRBs in FXS only includes males, whereas our sample encompasses the full FXS phenotype including both males and females, each of whom exhibit a different profile and trajectory of RRBs. Third, there may not have been significant findings for Restricted Interests, Compulsive behavior, Self-injury, or Ritualistic-sameness behavior because we examined them at the factor/subtype level rather than at the item level. Previous research suggests that specific items on RRB measures may differentiate individuals with FXS more so than subscales/subtypes (Abbeduto et al., 2019; Moss et al., 2009). For example, in past research only one individual item from the Insistence-on-sameness subscale of the RBQ – “just right” behavior – was significantly correlated with ASD symptomatology (Moss et al., 2009), suggesting that many RRBs commonly observed in individuals with FXS may not be associated with other symptoms of ASD (Oakes et al., 2016). Finally, the lack of significant main effects for Restricted Interests, Compulsive and/or Ritualistic-Sameness behavior may be accounted for by a lack of sensitivity in the RBS-R for detecting verbal perseveration, which tends to be highly elevated in FXS (Martin et al., 2012). Increased sensitivity in measures of RRBs in FXS may be necessary to detect a significant association between anxiety and verbal ritualistic behavior.

### Limitations and Future Directions

The present study had several strengths including our relatively large sample of youth with FXS (*N* = 60), the prospective longitudinal nature of the study, which is scarce in FXS, and the use of a validated factor structure in RRBs. However, there are several limitations. First, relying on parent-reported measures alone may possibly limit the scope of anxiety and RRBs detected in youth with low cognitive and/or verbal abilities, particularly in the case of anxiety that presents atypically (Ezell et al., 2019). That is, cognitive ability and communication skills may be limited in FXS and obscure anxiety or perceived RRBs, or may contribute to increased subtlety in some RRBs as well as manifestations of anxiety; thus, parents may have difficulty discerning whether certain behaviors relate to RRBs or anxiety. This was possibly compounded by using an anxiety measure normed on neurotypical youth (the CBCL). Although the CBCL has been widely used to assess anxiety in ASD (e.g., Baribeau et al., 2020) and FXS (e.g., Wall et al., 2019; Roberts et al., 2019a), some of the standard parent-report anxiety questionnaires designed for neurotypical populations (e.g., CBCL) have reduced sensitivity to detect clinical anxiety in ASD, particularly in children with ID (Kerns et al., 2021). Third, the lack of a comparison group limits our ability to determine the specificity of these findings to FXS. Finally, our study lacked purely objective measures of RRBs (such as wireless sensors that automatically recognize stereotypical motor movements; Goodwin et al., 2014), somewhat limiting our ability to fully differentiate RRBs within our measure of ASD severity. However, it is worth noting that the CARS captures a wide variety of phenotypic features of ASD, only few of which are specific to RRBs, supporting our findings that severity of ASD features contributes to the development of RRBs in some capacity. Further, as previously noted, RRBs are a transdiagnostic symptom in ID (Kästel et al., 2021).

To address these few limitations, future work should aim to include direct behavioral observations of RRBs and anxiety as part of a multi-informant, multi-method assessment to supplement parent reports and corroborate the findings from this study. This approach could capture the duration and intensity of RRBs in addition to the topography, which may better determine whether the *intensity* of RRBs, rather than presence/absence, in fact differentiates comorbid autism in individuals with FXS and potentially better differentiates anxiety from ASD in FXS. In addition, future research should employ measures that were developed to detect anxiety in youth with neurodevelopmental disorders, such as the *Parent-Rated Anxiety Scale for ASD* (*PRAS-ASD*; Scahill et al., 2019), which was recently created to reduce parents’ reliance on subjective, language-dependent items (such as “worries”) and focus more on observable behavioral manifestations of anxiety (e.g., hyperventilates when worried or afraid). Finally, future research should examine potential bidirectional effects between anxiety and RRBs in youth with FXS. After all, while we examined the contribution of early anxiety to later RRBs, some researchers have found that RRBs preceded the onset of anxiety in ASD and therefore conceptualize RRBs as early manifestations of anxiety (Baribeau et al., 2020; Baird et al., 2012) or as a “response to negative affect, which subsequently increases one’s risk of anxiety over time” (Sellick et al., 2021). Similarly, while we examined the contribution of ASD symptoms to later RRBs, it could be that high levels of RRBs may – at least in part – account for many of the ASD-like symptoms that are observed in individuals with FXS (Wolff et al., 2012). Thus, further longitudinal research is needed to disentangle the complex relationships between anxiety, autism symptoms, and RRBs in individuals with FXS.

## Conclusion

In sum, we found that anxiety and autism severity each contributed independently to RRBs at elevated levels of symptomatology. Overall, this study improves our understanding of the early risk factors contributing to later RRBs in youth with FXS. Moreover, our results inform the ongoing debate regarding the independence of anxiety and autism in individuals with FXS (e.g., Roberts et al., 2020; Wall et al., 2019). Although it is often presumed that increased anxiety causes or contributes to the misdiagnosis of ASD in FXS (i.e., the notion that high rates of anxiety make some children with FXS look like they have autism when they really do not), our findings suggest that anxiety and ASD appear to be distinct comorbidities in FXS that contribute to RRBs independent of one another. Ultimately, our findings contribute to an increased understanding of discrepancies in previous studies that implicated only anxiety *or* ASD in driving RRBs in FXS, given that we examined both anxiety *and* ASD symptomatology in FXS over time. Interestingly, we found that some types of RRBs – Sensory-motor behaviors – do not appear to be transdiagnostic across severe anxiety and ASD; rather, they appear to be more distinct to either anxiety or autism in youth with FXS, at least when at elevated levels.

Understanding the factors that predict RRBs in youth with FXS has important implications for the creation of targeted treatments for this population. While there is a dearth of intervention research that addresses RRBs in individuals with ASD (Uljarević et al. 2017), there are virtually no behavioral intervention studies that target RRBs other than SIB in youth with FXS (Moskowitz & Jones, 2015). Given our findings indicate that anxiety and autism represent two distinct pathways to certain RRBs in youth with FXS, possibly suggesting that RRBs might function to reduce anxiety/arousal for some children with FXS (anxiety pathway) and to obtain sensory input or increase arousal for other children with FXS (autism pathway), interventions that target RRBs in FXS should target these different mechanisms – that is, they should be function-based in an effort to maximize efficacy (Ingram et al., 2005). If anxiety represents a pathway to RRBs in youth with FXS, then interventions targeting anxiety should be incorporated into function-based treatments for youth with FXS who display RRBs and anxiety. Although there are only a handful of behavioral intervention studies that treat anxiety in individuals with ASD who have a co-occurring ID (Blakeley-Smith et al., 2021; Moskowitz et al., 2017; see Rosen et al., 2016 for a review), there have been *no* behavioral treatment studies for anxiety in individuals with FXS, which represents a significant gap in the literature and a critical area for future research. Overall, with an increased understanding of the role that anxiety and autism symptomatology play in the development of various types of RRBs in FXS, clinicians may be able to focus their interventions to address the true underlying or root cause of RRBs for a particular child.

## Figures and Tables

**Table 1:** Participant Demographics and Descriptives

Variable		
	Mean	SD
Total Participants, n	60	NA
T1 Age, months	59.35	48.87
T3 Age, months	92.25	46.12
Males, n (%)	48 (80.00)	NA
Full Scale Cognitive Score, SS	56.15	11.03
T1 CBCL Anxiety Mean Raw Score	0.36	0.30
T1 CARS Total Score	27.15	6.43
T1 RBS-R Bishop Factors		
Sensory Motor	5.53	3.62
Restricted Interests	1.40	1.59
Self-Injury	2.47	3.24
Compulsive	3.42	4.38
Ritualistic Sameness	4.91	5.16
T3 RBS-R Bishop Factors		
Sensory Motor	5.70	3.93
Restricted Interests	1.88	1.74
Self-Injury	2.70	3.37
Compulsive	4.05	4.72
Ritualistic Sameness	5.75	5.39

Notes. SS = Standard Score. CBCL = Child Behavior Checklist. CARS = Childhood Autism Rating Scale. RBS = Repetitive Behavior Scale - Revised (RBS-R)

**Table 2:** Moderated Regression Models

Sensory Motor Behaviors	*b*	*SE*	*p*
Intercept	5.89	0.42	< .001
Anxiety	−0.41	1.45	0.78
Autism Severity	0.25	0.08	0.002
T1 Sensory-Motor RRBs	0.39	0.13	.004
T1 Age	−0.01	0.01	0.118
Anxiety*Autism	−0.40	0.17	0.024
**Restricted Interests**	*b*	*SE*	*p*
Intercept	1.95	0.21	< .001
Anxiety	0.33	0.72	0.648
Autism Severity	0.06	0.04	0.117
T1 Restricted Interest Score	0.38	0.14	0.010
T1 Age	0.00	0.00	0.568
Anxiety*Autism	−0.13	0.08	0.117
**Self-Injurious Behavior**	*b*	*SE*	*p*
Intercept	2.77	0.39	< 0.001
Anxiety	0.02	1.35	0.986
Autism Severity	0.12	0.06	0.069
T1 Self-Injurious Behavior Score	0.49	0.13	< .001
T1 Age	0.01	0.01	0.587
Anxiety*Autism	−0.14	0.16	0.402
**Compulsive Behavior**	*b*	*SE*	*p*
Intercept	4.16	0.52	< .001
Anxiety	−0.92	1.95	0.639
Autism Severity	−0.01	0.09	0.954
T1 Compulsive Score	0.70	0.15	< .001
T1 Age	−0.00	0.01	0.786
Anxiety*Autism	−0.22	0.22	0.319
**Ritualistic Sameness**	*b*	*SE*	*p*
Intercept	5.91	0.63	< .001
Anxiety	−0.38	2.27	0.868
Autism Severity	0.03	0.11	0.774
T1 Ritualistic Sameness	0.53	0.16	0.001
T1 Age	0.01	0.02	0.419
Anxiety*Autism	−0.33	0.27	0.239
**Total RRBs**	*b*	*SE*	*p*
Intercept	20.65	1.56	< 0.001
Anxiety	−0.67	5.63	0.906
Autism	0.42	0.27	0.123
T1 Total RRBs Score	0.52	0.14	< 0.001
T1 Age	0.01	0.04	0.844
Anxiety*Autism	−1.19	0.67	0.081

## Data Availability

Data from the findings of this study are available from the authors upon request.
